# Supplementary figures and images for: The neglected giants: Uncovering the prevalence and functional groups of huge proteins in proteomes
Source: PLoS Comput Biol. 2024 Sep 16;20(9):e1012459. doi: 10.1371/journal.pcbi.1012459 (PMC11573180; doi:10.1371/journal.pcbi.1012459)

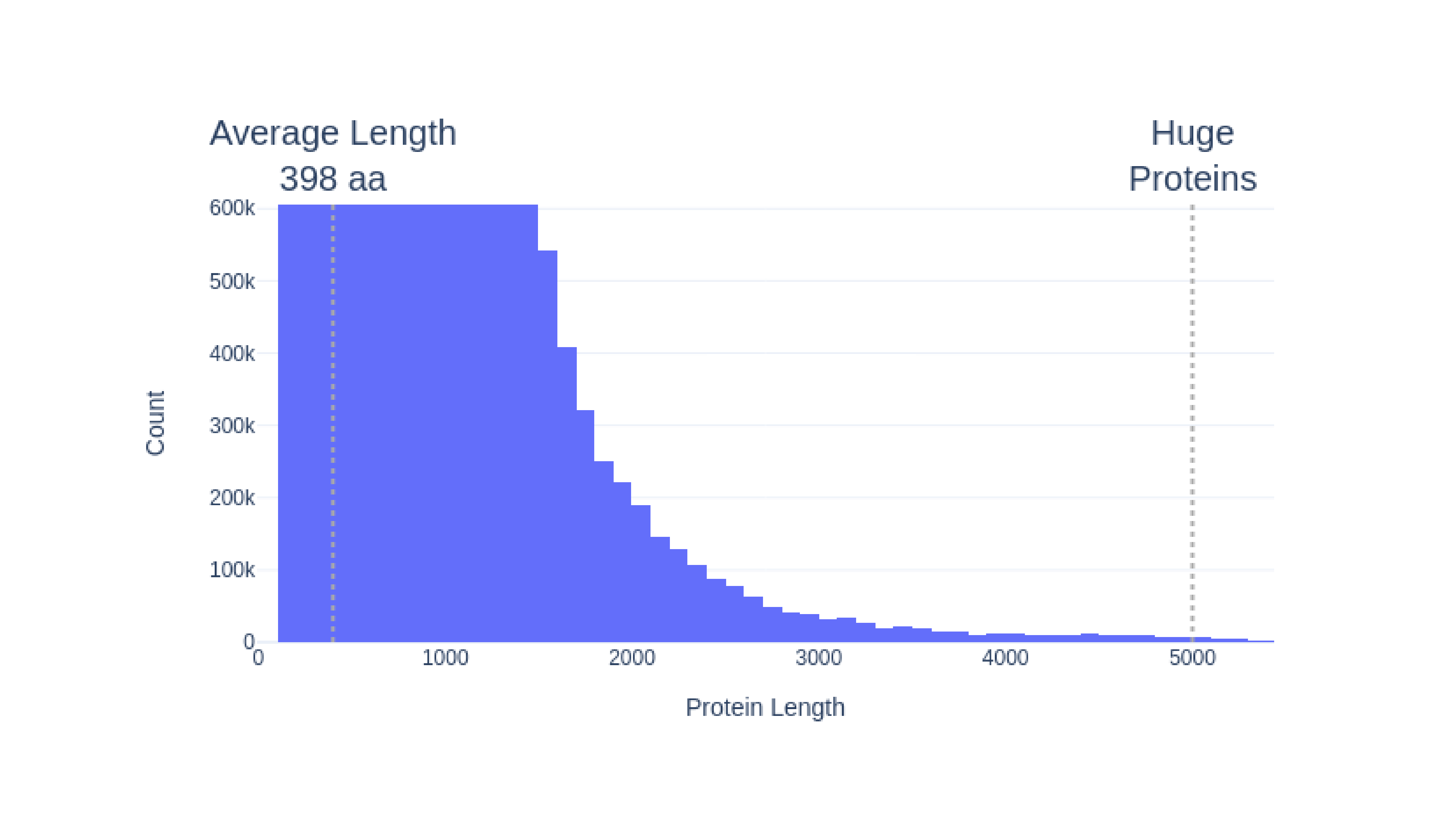

Supplement: S1 Fig — Showing the considerable number after the protein length average and before the 5000 threshold. (TIFF) [file pcbi.1012459.s001.tiff]

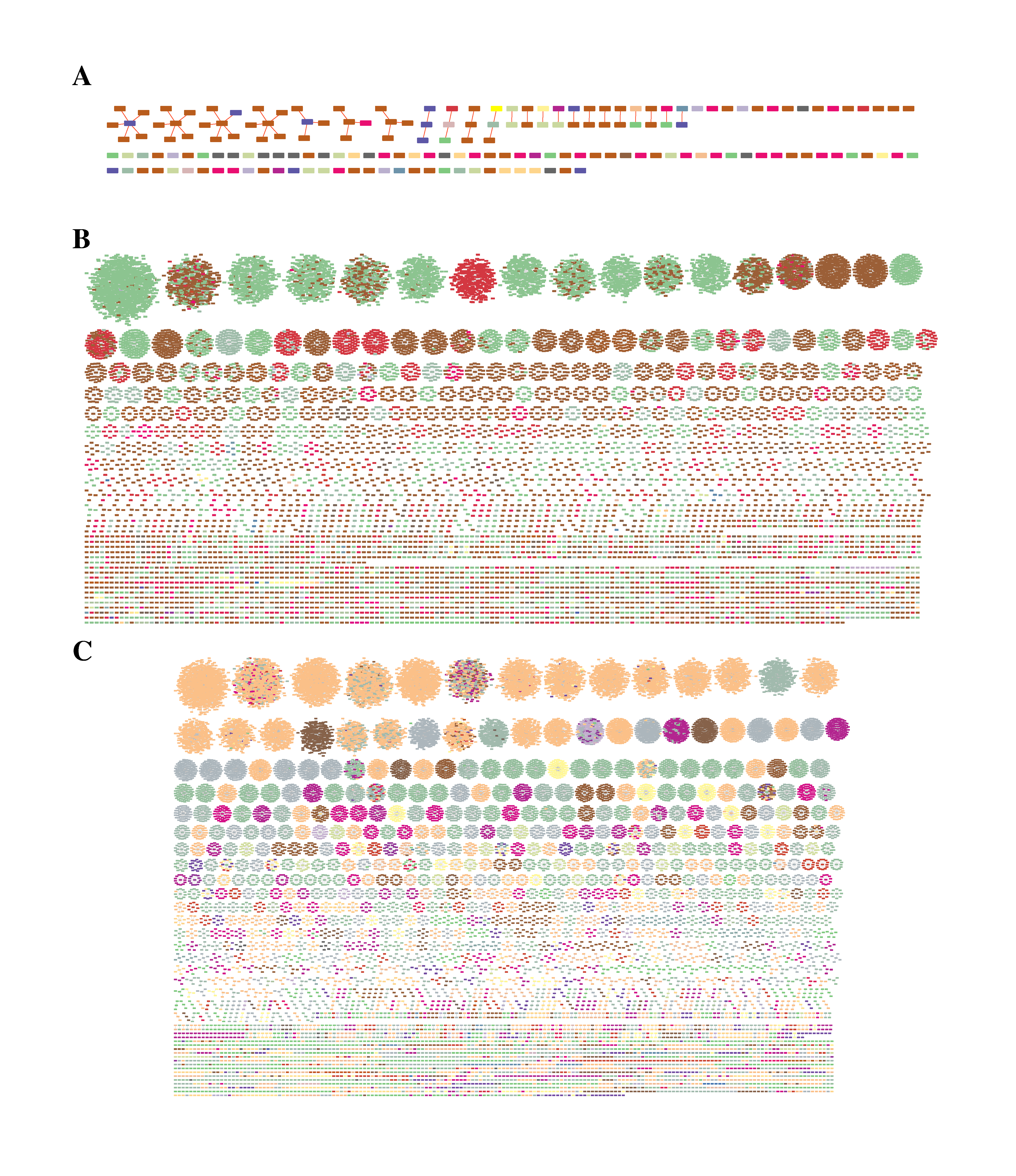

Supplement: S2 Fig — All clusters found colored by phyla, (A) Archaea, (B) Bacteria and (C) Eukaryotes. (TIFF) [file pcbi.1012459.s002.tiff]

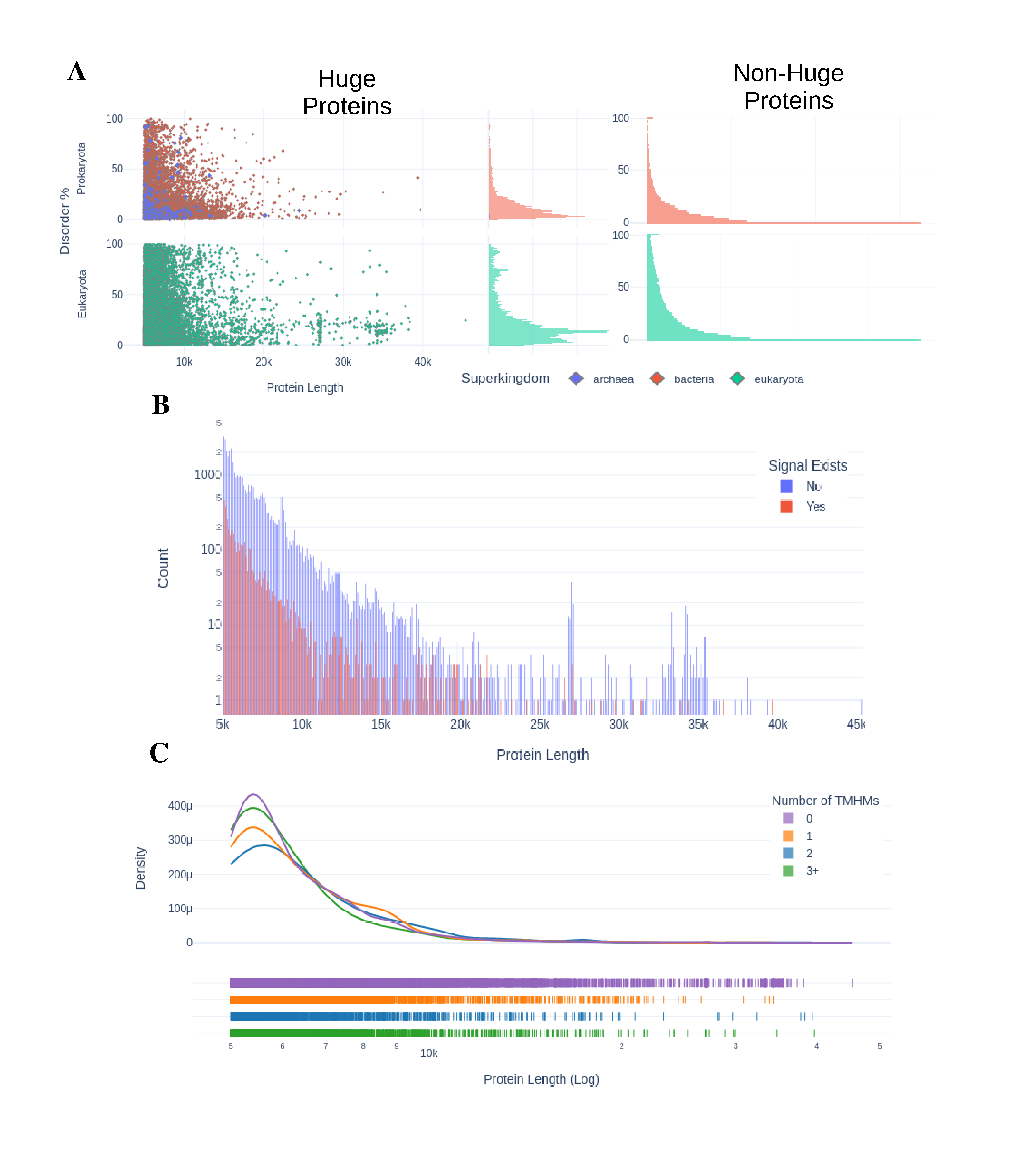

Supplement: S3 Fig — (A) Scatter plot the Disorder percentage in relation to Protein Length. On the right is the same analysis, for Non-Huge proteins (less than 5000 aa’s). From top to bottom divided between Prokaryotes and Eukaryotes, respectively. Each subplot is followed by a histogram of disorder percentages. (B) Histogram of presence (red) or absence (blue) of signal peptides in relation to Protein Length. The y-axis is in logarithmic scale. (C) Density plot of the distribution of number of Transmembrane helices (TMHs) in relation to Protein Length, with corresponding rug-plot. Colored based on number of TMHs. The x-axis is in logarithmic scale. (TIFF) [file pcbi.1012459.s003.tiff]

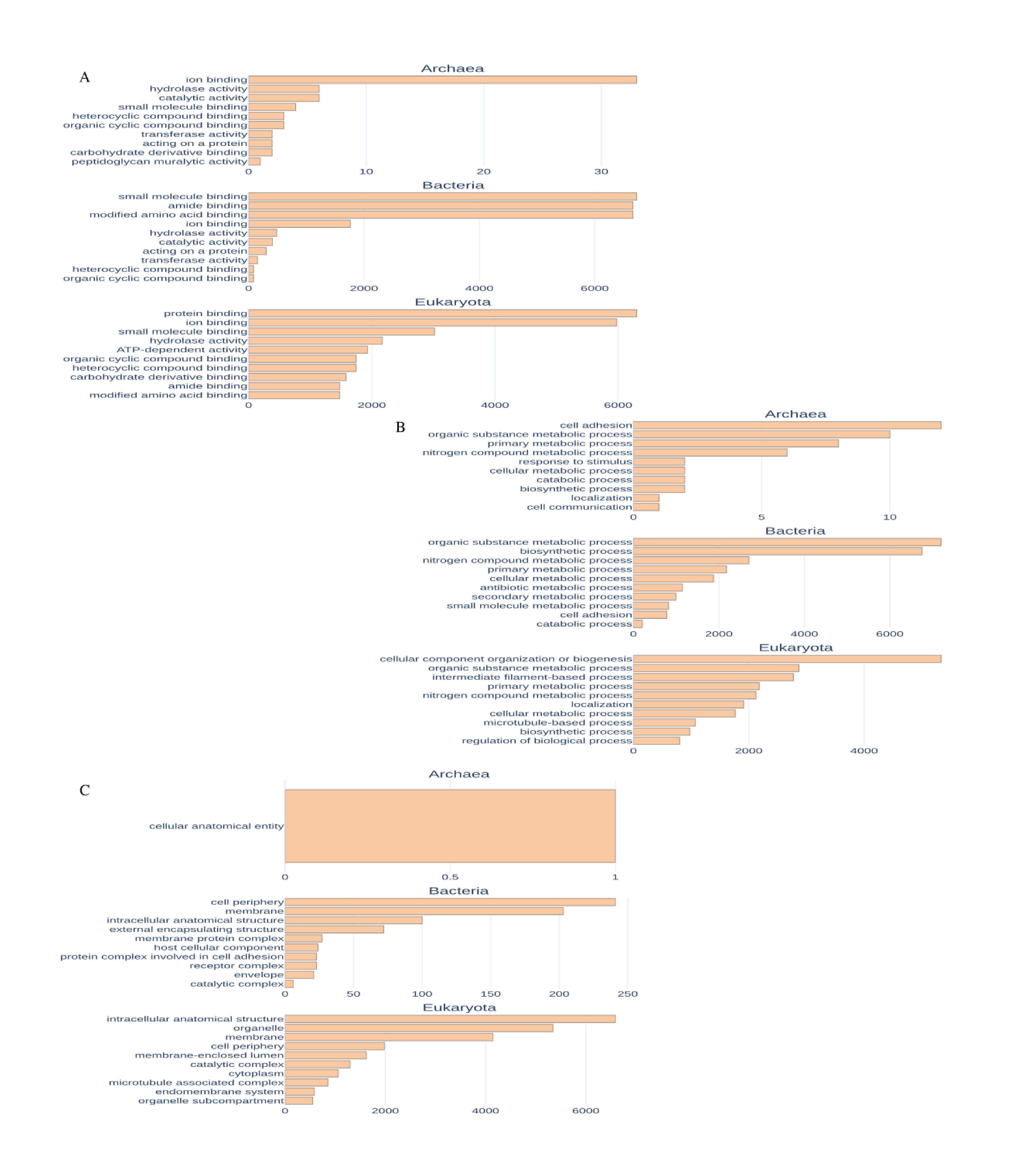

Supplement: S4 Fig — (A-C) corresponds to molecular function (MF), biological process (BP) and cellular component (CC), respectively. Each protein can contribute more than one GOterm to each category. (TIFF) [file pcbi.1012459.s004.tiff]
