# Supplementary material for: The neglected giants: Uncovering the prevalence and functional groups of huge proteins in proteomes
Source: PLoS Comput Biol. 2024 Sep 16;20(9):e1012459. doi: 10.1371/journal.pcbi.1012459 (PMC11573180; doi:10.1371/journal.pcbi.1012459)
Supplement: Attachment — Submitted filename: response_to_editors.pdf [file pcbi.1012459.s008.pdf]

Dear editors Erich and Arne,
we thank you very much for your consideration of our manuscript.
Please find below a detailed answer to your and the reviewer’s concerns, with our replies in bigger font. We have also uploaded a new version of the manuscript taking into account those comments.
Hoping that the new version of the manuscript satisfies your concerns,
Best wishes,

João and Damien

PLOS Computational Biology
Mon, Mar 11, 3:51 PM (8 days ago)
to me

CC: "Aníbal Segurilho Amaral" joao.17.anibal@gmail.com

Dear Dr Devos,

Thank you very much for submitting your manuscript "The neglected giants: Uncovering the prevalence and functional groups of huge proteins in proteomes" for consideration at PLOS Computational Biology.

As with all papers reviewed by the journal, your manuscript was reviewed by members of the editorial board and by several independent reviewers. In light of the reviews (below this email), we would like to invite the resubmission of a significantly-revised version that takes into account the reviewers' comments.

While the reviews seem quite clear and helpful overall, we would like to invite you to pay particular attention to their suggestions regarding the statistical support, checking at the nt level the data not being artifacts and the clarity of the presentation, including the writing and the legibility of figures.

R: We thank you and the reviewers for this. We have now done additional experiments to address these points and further concerns from the reviewers. Regarding statistical support we now state which methods were used and the corresponding results, adapting the text as needed. We conclude that the provided evidences already take into account data at the gene/genome level. We further improved the presentation, text for clarity and figures where suitable.

We cannot make any decision about publication until we have seen the revised manuscript and your response to the reviewers' comments. Your revised manuscript is also likely to be sent to reviewers for further evaluation.

When you are ready to resubmit, please upload the following:

[1] A letter containing a detailed list of your responses to the review comments and a description of the changes you have made in the manuscript. Please note while forming your response, if your article is accepted, you may have the opportunity to make the peer review history publicly available. The record will include editor decision letters (with reviews) and your responses to reviewer comments. If eligible, we will contact you to opt in or out.

[2] Two versions of the revised manuscript: one with either highlights or tracked changes denoting where the text has been changed; the other a clean version (uploaded as the manuscript file).

Important additional instructions are given below your reviewer comments.

Please prepare and submit your revised manuscript within 60 days. If you anticipate any delay, please let us know the expected resubmission date by replying to this email. Please note that revised manuscripts received after the 60-day due date may require evaluation and peer review similar to newly submitted manuscripts.

Thank you again for your submission. We hope that our editorial process has been constructive so far, and we welcome your feedback at any time. Please don't hesitate to contact us if you have any questions or comments.

Sincerely,

Erich Bornberg-Bauer

Guest Editor
PLOS Computational Biology

Arne Elofsson
Section Editor
PLOS Computational Biology

****

Reviewer's Responses to Questions

Comments to the Authors:
Please note here if the review is uploaded as an attachment.

Reviewer #1: The manuscript "The neglected giants: Uncovering the prevalence and functional groups of huge proteins in proteomes" by Amaral and Devos, investigates the phenomenom of large proteins beyond 5000 amino acids in length. A special emphasis of their study is on the "confirmation of existence" of those huge proteins through several proxy metrics to falsify is they are artifacts of coding predictions or other methods. This is a rather small, initial study on a yet understudied topic. I think its a fairly good approach of the authors to first examine if those proteins are not artifactual.

We thank the reviewer for this positive overview of our study.

Major:
- Please analyse how repetitive and complex the composition of these protein sequences are. This could give further indications on how they evolved.

R: Please also note that, as mentioned by the reviewer, this is a preliminary analysis, hoping to raise interest in this subset of proteins. We made no attempt to address evolution of these proteins at that point just yet. This is something we are considering for upcoming studies. Despite this, we indeed analysed the aminoacid content of these proteins (at the superkingdom level).

- Figure 2A and 2B, please conduct appropriate statistical tests on this data. Also, the low values in this Figure are hard to see and paritally covered by the orange-dotted box.

R: We have modified the figure in order to ease visualization. We addressed the concern about statistical support, showing that both Kruskal-Wallis and One-way ANOVA have a pvalues of 1.51e-171 and 1.20e-118, respectively, for the hypothesis that both have the same trend (figura 2A and B). Thus supporting this hypothesis. We attempted to show that this occurs due to the higher percentages of proteins with evidence at the protein level in HPs compared to Non-HPs. Thus further supporting their existence.

- Statistical values should be stated in the text of the result sections and not simply termed "slight positive correlation" etc., if statistical tests were actually performed. This is not clear to me from reading of the manuscript and is a major issue which will have to be fixed.
- Same problem lines 102-112

R: We modified the text to be more explicit. In the case of figure 2C, which refers “slight positive correlation” we show now in the figure all the correlation values between the variables tested. Meaning that genome completeness doesn’t have a major influence in the number of huge proteins found nor the length of these proteins. Although it doesn't support more huge proteins in more completed genomes, it in fact discard less complete genomes giving rise to more huge proteins. We hope that combined with the answer above, this satisfy the concerns of the reviewers.

- Generally, the text of the results section is very independent of the methods. Would like to have one short sequence for each result which tools are used. Or even only brackets. Now it reads like it comes all out of thin air

R: Done.

- line 167-182, please provide values.

R: Done.

Minor:
- Citation for the first claim (line 44)

R: Done.

- Sudden switch in text in lines 81-85. Feels deattached to the rest of the introduction.

R: Modified.

- Why exactly is the mere existence of such proteins surprising (line 117)? Please elaborate.

R: As we observed in the results of the first analysis/figure1 A, these proteins are clear outliers of the protein length distribution. Mostly due to the fact that the probability of not encountering a stop codon decreases exponentially with the length of a sequence.

- Fig 3A and 3B, numbers in the legend for proteins in those cluster would be helpful

R: Done.

- Line 206, please elaborate how these huge proteins could be novel ones? Novel or homology-lacking proteins were yet mostly shorter proteins or rather peptides. In my opinion, this could be an addtitional and interesting point of this study.

R: We state that there is the possibility of these proteins being novel ones, just because we cannot discard this based on the observations. Although, for this particular subset, the most likely scenario is that they are just artifactual.

- Please add the phyla to your Supplementary table 2

R: Done.

- Some Figures are hard to read (very small font) and seem a bit overstretched e. g. Figure 1D

R: We have modified the small font and more, hoping that this improved the reading.

Reviewer #2: This is a potentially very interesting piece of work, investigating the extremes in terms of open reading frame length. At the outset I very much wanted to like this work, and I remain supportive. But I think there is a little too much optimism here and some additional due diligence to ensure that we are not just observing database issues needs to be done. In particular there is no attempt to use nucleotide data to support that the predicted ORFs are actually real (nucleotide frequencies can be very different between expressed regions and intergenic sequences for example). I also felt that the discussion does not fully expose the possible interpretations and evolutionary models - the Huntington's example seems to me to be a bot silly when dealing with such disparate sizes. The obvious interpretation from the examples given in F4 would presumably be expansion or fusion of an array of paralogues as four of the examples are clearly repetitive. Specific comments are below;

R: We thank the reviewer for the constructive feedback. Our main objective with this study was to report the interesting outlier sequences that we found. Thus, one of our main concerns was to determine if these were artifactual (figure 2). We think that the analysis of Uniprot Protein Existence Levels, the BUSCO completeness of the genome and the "Assembly level " of the genome, already incorporates in parts the nucleotide evidence. We agree that the Huntington's example might be too far reaching and thus we removed it. Also we added the hypotheses of expansions and fusion of paralogues as suggested.

Advances in biology often arise from the exploration of extremes.
Sweeping statement and not clear it is true.

R: Clarified.

5.000 amino acids

Odd; usually either 5 000 or 5,000? As is this means five…

R: Agree, changed.

majority found in Eukaryotes

Frequency per million ORFs?

R: Figure 1D shows that the majority of datapoints (proteomes) are in the Eukaryotic section (green) thus suggesting a higher number of huge proteins (and by proxy ORFs) in Eukaryotes.

Abstract and summary are very similar

R: Changed summary.

Alternate between colossal, massive and huge. Suggest decide on one term throughout?

R: Changed to use “huge” throughout the text.

L66 Nonribosmal

R: Modified.

L87 Where do they occur?

R: Changed the title of the section.

F1B Need to make clear this plot starts at 5k. Suggest in A use a different color to avoid confusion?

R: Added 5k mark at the start of the x-axis in the inset, also changed the color of the figure 1A to grey, to not be confused with the blue from Archaea in the inset.

F1D Seems a missed opportunity to not include some Asgard Archaea?

R: We agree that the suggestion would be interesting. Despite this, the analysis was done at the phyla level, and the suggestion would require us to include other clade-level groupings in order to include Asgard. We removed phyla with less than 5 proteomes, meaning that in the dataset there are some Asgard Archaea proteomes but were removed by the filtering step in an effort not to skew the interpretation.

F2A I think this needs better explanation as this seems a bit odd to me. The idea that less than 1% of proteins in the databases have evidence of existence at transcript level seems very low or am I misreading this. I’m also unclear what homology means in the present context - is this a single domain or across the entire length? And in this case what does predicted mean - simply what is in database but lacks other evidence? And finally bin 5 seems empty; please explain.

R: This visualizes the level at which each protein is annotated regarding the type of evidence that exists, where only the most solid evidence is reported. If there is evidence at protein level this supersedes evidence at the transcript level even though it might exist. According to Uniprot: “Only the highest or most reliable level of supporting evidence for the existence of a protein is displayed for each entry. For example, if the existence of a protein is supported by both the presence of ESTs and direct protein sequencing, the protein is assigned the value 'Experimental evidence at protein

level'." These two links further illustrate the point: https://www.uniprot.org/help/protein_existence and this https://ftp.uniprot.org/pub/databases/uniprot/current_release/knowledgebase/complete/docs/pe_criteria.txt.

How accounting for erroneous intron predictions?

R: We have not attempted to look at introns as this would only apply to eukaryotic porteins, and its influence should be minimal. Besides this is somehow implicit in some data, e.g. Figure 3B shows a large number of eukaryotic sequences clustering, as it is unlikely that the same mistake would be made in many different sequences.

What is the quality cutoff for data concerning transcripts/protein detections? Obviously this is somewhat arbitrary but I think this needs to be in the main text at least in brief. Methods are rather brief.

R: We used all proteins present in UniprotKB, so the same standard was used. Expanded methods, also adding more detail to clarify some previous comments.

L149 Thus, the existence of HPs can be, indirectly and for the most part, linked to higher genome quality.

Grammatically this is very confusing.

R: Clarified.

F3 What is cut-off for largest clusters? Appear to be different genomes sampled for Archaea here to F1. Is this the case? And also for panels A and B? Not sure the point of panel D. I notice that the data in B are heavily Opistokhonta biased. Is there a reason for this? My bacteria taxonomy knowledge is not good enough to know how well the sampling here is - comment?

R: We only show the largest clusters for a visual representation, as we have over 2400 clusters. Clusters were filtered by superkingdom, sorted by size. We manually chose the top 30-35 largest. No cutoff was done in Archaea. Regarding the disparity between F1 and F3, in terms of Archaea genomes used, in F3 we didn't perform any filtering on the number of genomes in each phyla and we didn't add new phyla if it was lacking Huge proteins, contrary to F1.
We removed Panel D to avoid confusion, as it does not add information that needs to be conveyed through a figure.
Commenting on apparent bias towards Opisthokonta, this has two main reasons: first, there is an inherent bias in the sequence databases for higher quality genomes and proteomes of this clade. The second reason, more specific to this analysis, is a consequence of the first, has the largest clusters have higher representation of this group.

L167 The majority of Eukaryotic clusters are related to key intracellular processes.

Pretty true for the majority of any eukaryotic protein?

R: Yes, agreed. Changed the text to not state the obvious.

In F3 and T1 I am not seeing any attempt at statistical analysis. Also, as these proteins are huge, how many domains are there that are predicted for each? Point being that as there are possibly many domains the connection with a specific functionality may be difficult if, for example, domains that participate in different pathways are predicted as present in the same ORF. I'd really like there to be a more detailed analysis of at least a few examples, where there is confident evidence for expression. Appreciate this is in part in F4, but more examples would help.

R: Here we now state the values used for mmseqs clustering. Our goal with this figure was not to study each cluster from a network theory perspective(and its related statistics), but more to easily visualise each cluster. The clusters chosen were the bigger ones for each superkigdoms, besides

Archaea that we show all. Regarding the suggestion of a few examples with evidence for expression, we were able to find in our dataset a selection of proteins that have expression data associated. These include: Bltp1 from *Mus musculus* (Uniprot: BLTP1_MOUSE) that is expressed in several tissues at different developmental stages (ExpressionAtlas: ensmusg00000037270*).* Of course, from the Uniprot Existence Levels assigned, we find at least 26 huge proteins with "Evidence at transcript level", meaning they have associated expression data.

I don't really understand the basis for the 'Unreal' category. This seems to be a statement of the obvious in that some of these predictions are going to be incorrect. Many gene annotations are known incorrect due to our lack of complete understanding of splicing, initiation and termination signals.

R: We present these particular findings in the hope of improving the current sequence databases. We suggest the possibility of refining of the specific stated sequences, or just by further supporting the use of these methods to eliminate erroneous ones. Changed the title of this section to "Potential Artifacts" to be clearer.

F4 The prominence of clear domain repeats is not discussed. It is of specific interest as to how common the feature is across the HPs as it suggests a possible evolutionary model.

R: We further analysed the sequences with pfam domain assignment and found a total of 15400 proteins with domains label as "repeats", representing around 40% of all the proteins with pfam assignments (38248). This result hints at a possible evolutionary trajectory of these sequences, through the accumulation of repetitive motifs. Added to the text.

L267 As expected, the majority belongs to Eukaryotic proteomes, followed by Bacteria and very few representatives from Archaea

But this is a result of the bias in taxon sampling? Needs to be keyed to frequency?

R: Figure 1D show that this is not a result of bias, as it shows the number of HP normalized by "size of proteome", not the number of proteomes in a phyla (which would be frequency as proposed here). Overall, it could be interesting to key to frequency, but we don't believe it would change the overall trend.

Reviewer #3: This study describes a comprehensive bioinformatic analysis of large proteins or "huge proteins", those proteins exceeding 5,000 amino acids, which are often overlooked in biological research. The authors generated a set of 41,000 of these huge proteins from UniprotKB and then analyzed different aspects, including prevalence in difference organisms, likelihood to exist, clusters of orthologs, functions and functional domains, and abundance of structural features such as protein disorder and transmembrane helices.
The study makes several interesting observations. For example, huge proteins are often involved in key cellular processes, such as cytoskeleton organization and functioning as chaperones or E3-ubiquitin ligases in Eukaryotes, where in bacteria they primarily contribute to non-ribosomal peptide synthesis, polyketide synthesis, and play roles in pathogen-host interactions. The study underlines the importance of further exploring these giant proteins for their cellular roles, ecological significance, and potential biotechnological applications.

Overall, this is a well-written and interesting study addressing an unexplored topic. Below I include comments and suggestions to improve this work.

R: We thank the reviewer for the positive feedback and interesting observations.

The observation in lines 195-196 is intriguing: "Around 1442 proteins have no homology inside our dataset, but are assigned to COGs, suggesting a possible recent fusion of smaller proteins into HPs." Is there a computational method to verify this hypothesis, possibly through domain alignments?

R: The objective of this work was to report on these findings rather than resolve an evolutionary hypothesis of their origins. However, we think that COGs assignment means some similarities between proteins in these COGs and thus an evolutionary relationship.

Could the authors consider classifying those proteins not found in COGs by alternative methods? For instance, identifying common domains? Previous studies by the same group have uncovered important evolutionary relationships beyond sequence similarity. I could imagine that the standard definitions of orthologs using sequence similarity alone could potentially not capture distant biological relationships and even do this analysis on the whole set including on proteins with assigned COG.

R: Both the PFAM domain analysis and even more the clustering using mmseqs allowed us to classify these proteins without COG assignment, to a certain extent. In a future study a possible course of action would be to predict the structures through AlphaFold and then align them using Foldseek to cluster based on structure similarities. Although discussed, this was beyond the scope of this study, in particular due to the sheer size of these proteins and the size limit in AF2.

The COG annotations listed in Table 1 are quite general. A more detailed categorization could be beneficial.

R: Our objective with using COG annotations was to have broad groupings of functions for these proteins, in hopes of finding larger functional patterns despite the superkingdom. Besides, we also tried with GO terms to do a more detailed grouping (as presented in supplementary figure 4).

The analysis of various structural features seems brief, and I think it can add more to the study. Perhaps additional features should be included in the analysis (e.g. coiled coil regions, protein protein interactions). Further, the presence of a 30% disordered structure in a 10,000 amino acid protein warrants a deeper examination. Are these proteins characterized by more disordered domains, do they consist of numerous linkers, or do they correspond to the coiled coil type? The illustration in Figure 4 seems to indicate a combination. As recent studies reveal, disordered regions come in different forms. Due to the high abundance of disordered regions in huge proteins and their likely importance additional analysis should be conducted.

R: We agree that 100% disorder is suspicious (or having no disorder at all even more), and regards further analysis but is outside the scope of this paper. In addition, this is only a relatively small subset of the huge proteins, 15 with 100%, and 152 with 0% out of 41 754 huge proteins.

Corrections for several typos:

Line 72: change 'where' to 'were'
Line 151: change 'Fig3' to 'Fig 3'
Line 363: change 'this' to 'these'
Line 393: replace '. . ' with a single period '.'

R: All these typos were corrected.
