# Supplementary material for: The neglected giants: Uncovering the prevalence and functional groups of huge proteins in proteomes
Source: PLoS Comput Biol. 2024 Sep 16;20(9):e1012459. doi: 10.1371/journal.pcbi.1012459 (PMC11573180; doi:10.1371/journal.pcbi.1012459)
Supplement: Attachment — Submitted filename: response_to_editorsF.odt [file pcbi.1012459.s009.odt]

Dear editors Arne Elofsson,
Thank you very much for your work on our manuscript, as well as to the reviewers for their comments that have substantially improved our manuscript.
Having taking them all into account, we hope our manuscript is now acceptable for publication.

Best wishes,
João and Damien

-----

Dear Dr Devos,

Thank you very much for submitting your manuscript "The neglected giants: Uncovering the prevalence and functional groups of huge proteins in proteomes" for consideration at PLOS Computational Biology. As with all papers reviewed by the journal, your manuscript was reviewed by members of the editorial board and by several independent reviewers. The reviewers appreciated the attention to an important topic. Based on the reviews, we are likely to accept this manuscript for publication, providing that you modify the manuscript according to the review recommendations.
Please adress comments from Reviwer 2 before submitting the final version.
Comments to the Authors:
Please note here if the review is uploaded as an attachment.
Reviewer #1: The authors have addressed my concerns.
Reviewer #2: The majority of proteins have a length between 100 and 500 aminoacids (aa), approximately [1].

Not clear how a ramge is approximate.
R: We removed the approximately

presence of numerous proteins exceeding 5000 amino acids in length

Previous sentence has aa as abreviatio for amino acids, so use it.
R: Changed.

still Huge genes and huge genes
if using an abbrev then use it….
R: There is only one occurrence of huge genes in the text, so we didn’t think it necessary to include an abbreviation. If the reviewer is suggesting it for huge proteins, then we made sure to use the abbreviation where possible. 

Drosophila Melanogaster. ooops
R: Changed to “Drosophila melanogaster”
_
_has hardly been investigated
better - not thoroughjly
R: Changed the sentence to: “However, their role in the cell has not thoroughly been investigated.

where the organism is not growing
better - not proliferating
R: Changed.

This protein, with almost 10 000 aa, has been found to bind Fibronectin

fibronectin
R: Changed to lowercase.

and the recent descriptions of Omnitrophota species which have been found to
possess many HP some of which are the largest ever reported, even larger than eukaryotic one

and recent descriptions of Omnitrophota species which possess many HP, including some of the largest ever reported, exceeding the size of HPs suggested for eukaryotes.
R: Changed.

However, recent broad analyses are missing.

So????? What to address?
R: Clarified the statement.

we decided to answer a broad range of questions

address better
R: Agreed, address is better. “We decided to address a broad range of questions.

I have not put in additional issues in the wroting and hope that the authors will edit the MS carefully.
R: Further improved on the writing throughout the document, to help the flow and to correct certain spelling or grammar mistakes.
